# Precision Epidemiology: A Computational Analysis of the Impact of Algorithmic Prediction on the Relationship Between Population Epidemiology and Clinical Epidemiology

**DOI:** 10.3389/ijph.2024.1607396

**Published:** 2024-10-01

**Authors:** Elena Esposito, Paola Angelini, Sebastian Schneider

**Affiliations:** ^1^ Faculty of Sociology, Bielefeld University, Bielefeld, Germany; ^2^ Department of Political and Social Sciences, University of Bologna, Bologna, Italy; ^3^ Settore Prevenzione Collettiva e Sanità Pubblica Regione Emilia-Romagna, Bologna, Italy; ^4^ Human Media Interaction, Faculty of Electrical Engineering, Mathematics and Computer Science, University of Twente, Enschede, Netherlands

**Keywords:** precision medicine, algorithmic methods, statistical methods, population epidemiology, clinical epidemiology, precision public health, algorithmic prediction, topic modeling

## Abstract

**Objectives:**

Precision Medicine (PM) uses advanced Machine Learning (ML) techniques and big data to develop personalized treatments, but healthcare still relies on traditional statistical procedures not targeted on individuals. This study investigates the impact of ML on epidemiology.

**Methods:**

A quantitative analysis of the articles in PubMed for the years 2000–2019 was conducted to investigate the use of statistical methods and ML in epidemiology. Using structural topic modelling, two groups of topics were identified and analysed over time: topics closer to the clinical side of epidemiology and topics closer to the population side.

**Results:**

The curve of the prevalence of topics associated with population epidemiology basically corresponds to the curve of the relative statistical methods, while the more dynamic curve of clinical epidemiology broadly reproduces the trend of algorithmic methods.

**Conclusion:**

The findings suggest that a renewed separation between clinical epidemiology and population epidemiology is emerging, with clinical epidemiology taking more advantage of recent developments in algorithmic techniques and moving closer to bioinformatics, whereas population epidemiology seems to be slower in this innovation.

## Introduction

Recent developments in machine learning (ML), together with the production and use of large amounts of data of different forms (labeled as big data), are having a profound and complex impact on scientific research [1]. In the field of healthcare, Precision Medicine (PM) uses advanced digital techniques for the purpose of developing personalized treatments for individual patients.

The possibility of developing personalized medical treatments is certainly very attractive. Our familiar medical procedures, however, target all patients, albeit imprecisely, and our healthcare system is organized accordingly. The introduction of a personalized approach can have disruptive effects on the social reference of medicine, raising complex issues of compatibility with the demand for solidarity [2] - between more precise benefits for single patients and less accurate benefits for everyone.

Our research explores this dilemma by focusing on epidemiology, the discipline that in the medical field makes the greatest use of statistical procedures and stochastic mathematical modeling. We address the following research questions: how has the work of epidemiologists changed as a result of the availability of advanced machine learning techniques? How do they combine the “macro” aspect, addressing the population, with the “micro” aspect, addressing individuals? How has the role of epidemiology shifted relative to other biomedical disciplines and research areas outside the medical field (particularly bioinformatics)? With what consequences for the issues and purposes of epidemiological research?

The work offers insights derived from a qualitative examination of the evolution of epidemiology from a sociological perspective and, at the same time, employs a quantitative approach, utilizing natural language processing to analyze the field of epidemiological research. Our study uses a fitting structural topic model applied to an extensive corpus comprising 67,838 abstracts within the domain of medicine. Through this model, we systematically trace the temporal evolution of thematic patterns in epidemiological research.

### Collective and Individual Reference in Epidemiology

In the field of epidemiology, the challenges posed by personalization are combined with complex issues regarding the relationship between the collective and the individual dimension, which have accompanied the entire history of the discipline and touch upon its very nature and the definition of its tasks. A standard definition of epidemiology is: “the study of the distribution and determinants of health-related states or events in specified populations, and the application of this study to the prevention and control of health problems” [3]. To inform prevention and control, epidemiology adopts quantitative methods and in particular statistical tools. “Through a constantly evolving collection of cohort studies, randomised trials, and case-control studies, decades of epidemiological research have worked to quantify the nature and magnitude of associations between risk exposures and outcomes in studied populations” [4].

The definition of epidemiology presented above, like alternative ones, leaves a fundamental question open: how do we specify the population? Does one refer to the community as a whole or to narrower groups within it, limited to people at higher risk for various reasons? If the focus is on the broad population, the “prevention and control of health problems” will concern collective efforts organized by society to promote general health, targeting poverty, inequality, social and cultural determination of disease, pollution. Relevant expertise involves sociology, economics, behavioral and environmental science, media communication and policy making [5]. If the focus is on small groups, by contrast, prevention will address physiological and psychological determinants of disease, targeting lifestyle and metabolic factors like blood pressure, body mass index or gene mapping, or environmental exposure. Therefore, the contribution of clinicians, geneticists and molecular biologist becomes prevalent. Public health strategies, priorities and disciplinary positioning are different in the two cases.

In the first half of the 1900, epidemiology tendentially chose the first path, adopting a *macro* approach aimed at the population as a whole. Research in population epidemiology was focused on social and economic factors contributing to the spread of infectious diseases such as cholera and malaria or of conditions such as rickets and scurvy, whose control depends on water sanitation, food hygiene, sewage draining and disposal, and housing conditions. “Immense changes occurred during the second half of the 20th century. There was a shift of emphasis to non-communicable diseases, which reflected the decline of many infections and an increase in heart disease and cancer in many countries” [6]. The focus shifted from community ecology to *individual risk factors* and individual patients, bringing epidemiology closer to clinical practice. Methods and principles of epidemiology were applied to support decision-making in the management of individual cases. The expression “clinical epidemiology” was introduced at that time, to indicate a basic medical science devoted to “the application, by a physician who provides direct patient care, of epidemiological and biostatistical methods to the study of diagnostic and therapeutic processes in order to effect an improvement in health” [7]. The approach led to evidence-based medicine (EBM) which aims at “integrating individual clinical expertise with the best available external clinical evidence from systematic research” [8]. Here the goal is the care of individual patients, and the evidence is obtained with statistical tools, primarily the extremely powerful and successful RCTs (Randomized Control Trials, or in this context rather Randomized Clinical Trials).

The difference between population epidemiology and clinical epidemiology was already based on the distinction between general (macro) and individual (micro) reference, i.e., on the issues brought to the fore by the recent development of precision medicine, which has a specifical history in the field [9]. Since the 1980s, the debate within epidemiology highlighted the possible contrast between the two approaches. According to Last [10] the term “clinical epidemiology” is an oxymoron. By definition, in fact, epidemiology addresses a population, maybe reduced when it is a restricted population of patients rather than a community-based population. The CDC states that “In epidemiology, the patient is the community and individuals are viewed collectively” [11]. In Last’s opinion, therefore, the object of epidemiology cannot be individual persons as in the case of clinical epidemiology, because this would call into question the identity of the discipline and its research agenda linked to community-based preventive medicine and general health promoting activities.

Does then the contrast between individualization and generalization in PM introduce no real novelty in the field of epidemiology, besides the further sophistication of the quantitative techniques used by the discipline? Our research explores an alternative possibility, referring to the innovative aspects of recent algorithmic techniques.

### What Is the Focus of Precision Epidemiology?

Prior to ML, the statistical techniques and tools used in population epidemiology and clinical epidemiology were largely the same. The second half of the twentieth century had witnessed “the victory of statistics” [12–14]. The difference between the macro and the micro approach is that statistical tools refer in the former to data on a multiplicity of individuals (the population) and in the latter to narrower groups or even to single individuals (up to N-of-1 or single subject trials [15–18]).

In the last decades of the 20th century, epidemiology, biomedicine and clinical practice gradually came closer. RCTs became the standard testing procedure in all three fields. In cancer medicine and cardiology, for example, statistical designs of clinical trials created the very platform for clinical experimentation [13, 14, 19], and epidemiology became a core discipline in cancer control [20], (setting the agendas of public campaigns of cancer prevention [21, 22]). Epidemiology handbooks introduce both approaches seamlessly [11, 23].

Within this framework, the approach of PM might introduce a more radical discontinuity [24–26]. Models using ML algorithms can identify multifaceted non-linear patterns in large amounts of unsampled and uncontrolled data without a driving hypothesis, only searching for regularities that may anticipate future trends. For epidemiological research this makes a big difference. Researchers using machine learning have begun to explore heterogeneous treatment effects instead of overall average effects [27, 28]. Machine learning models can evaluate many variables to define heterogeneous effects without increasing statistical error, detecting very specific clusters of individuals who show different treatment effects [29]. The promises of PM are based on these opportunities.

But epidemiology, as we have seen, is defined precisely by its focus on population. Is this changing and how, in the era of precision medicine and machine learning? In epidemiological research using big data and machine learning, the chronic opposition between micro and macro dimensions might be entering a new evolutionary stage. Is this affecting the methodological approach and the distribution of goals and methods between population epidemiology and clinical epidemiology? Are the relationships with other disciplines changing? The next section of our paper explores these questions empirically.

## Methods

### The Impact of Algorithmic Techniques on Population Epidemiology and on Clinical Epidemiology

To investigate these issues, we carried out a quantitative analysis of a dataset of articles in the years 2000–2019 collected on PubMed. We decided to restrict our analysis to articles published until 2020 because of the impact of the pandemic on research and on publications in population epidemiology, combined with the exponential growth in the use of ML and other AI techniques in recent years. The procedure used in our analysis is presented in detail in the Supplementary Appendixes. Here we describe the main steps of our investigation and their results.

We queried PubMed for research articles on epidemiology in journals in medicine, epidemiology, bioinformatics, and artificial intelligence in medicine, using the MeSH (Medical Subject Headings) terms for epidemiology and epidemiological methods. MeSH (Medical Subject Headings) terms are a standardized vocabulary indexing biomedical literature, ensuring precise and efficient searches in databases like PubMed. These terms are hierarchically organized and include synonyms to cover variations in terminology. Automatic term mapping enhances search accuracy by converting common language phrases into MeSH terms, ensuring comprehensive retrieval of relevant articles and improving search consistency. We used MeshTerms to query PubMed without specifying which of the Mesh Terms belongs to which journal (and thus to a specific research community). We found that Mesh Terms associated with machine learning and those associated with statistics were used in distinct journals. In general, standardization reveals areas of specialization, research trends, and interdisciplinary collaborations, making it easier to identify and differentiate between fields such as public health and clinical medicine. Additionally, MeSH terms streamline literature searches and assess journal scope. To distinguish between different methods, we used MeSH terms for methods associated with machine learning and for “classical” statistical methods (see Supplementary Appendix SA.1.1.1. for the query strings). The query results exceeded 1 million publications; we decided to restrict our analysis to the most important journals. We identified the top journals on medicine and health, epidemiology, computational biology, and medical informatics using Google scholar (https://scholar.google.com/citations?view_op=top_venues&hl=en; see Supplementary Appendix SA.1.1.4).

**FIGURE 1:**
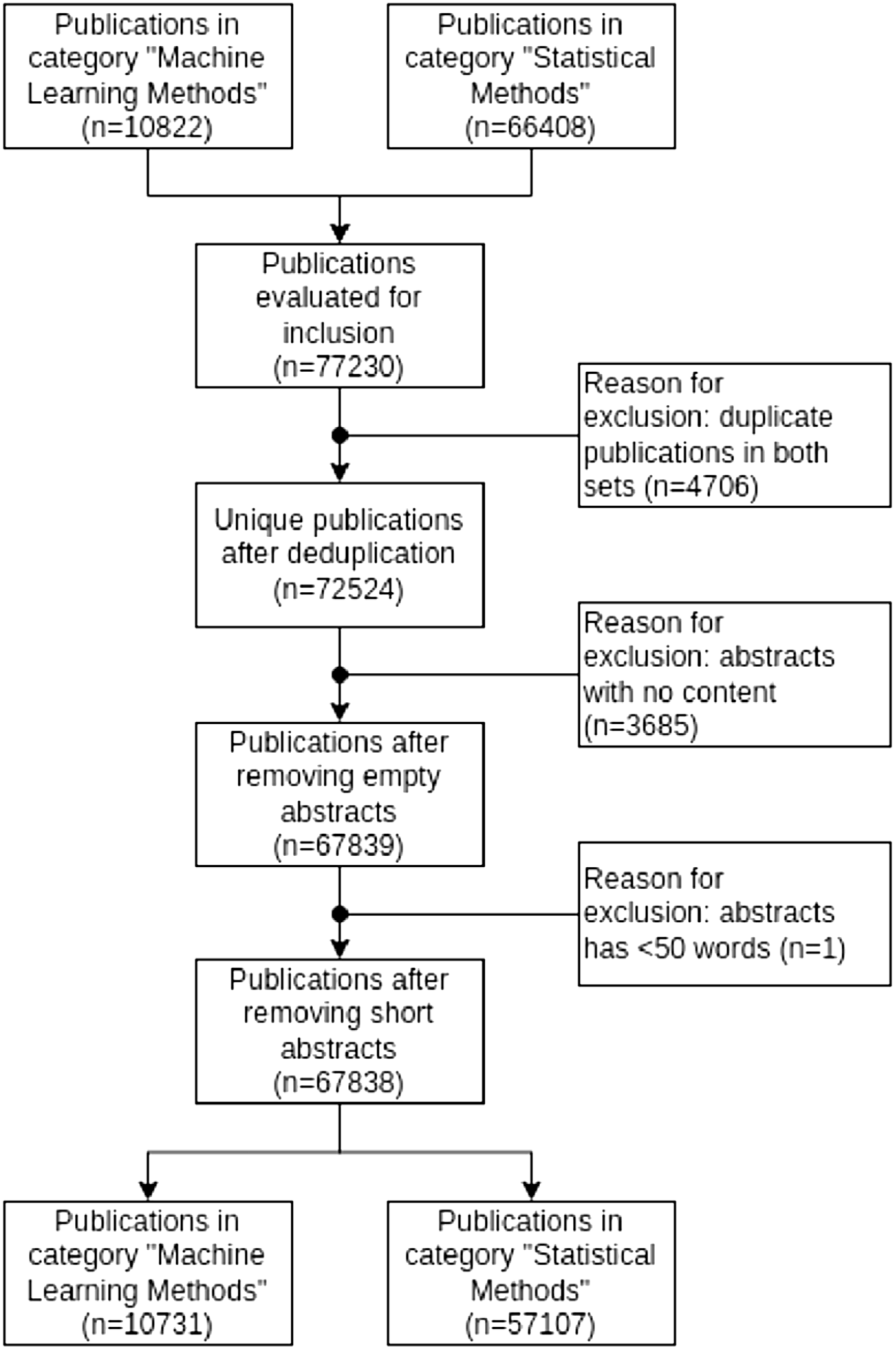
Flow chart explaining the inclusion and exclusion criteria for structural topic modeling (Germany, 2023).

The queries resulted in 10,822 publications for machine learning methods, 66,408 publications for statistical methods (5,706 overlapping publications). We cleaned up our results excluding first duplicate publications in both corpora, then publications without abstracts and finally publications whose abstract was shorter than 50 words (that would not be suited for an exploration of the dataset with unsupervised topic modeling). Figure 1 presents a flow chart explaining the inclusion and exclusion criteria to select the publications for our analysis.

We have collected N = 67,838 publications in total, including abstracts, authors, keywords, departments, and year of publication for further inspection. These publications produced again two corpora, distinguishing publications using machine learning methods (n = 10,731) and publication using statistical methods (n = 57,107).

**FIGURE 2:**
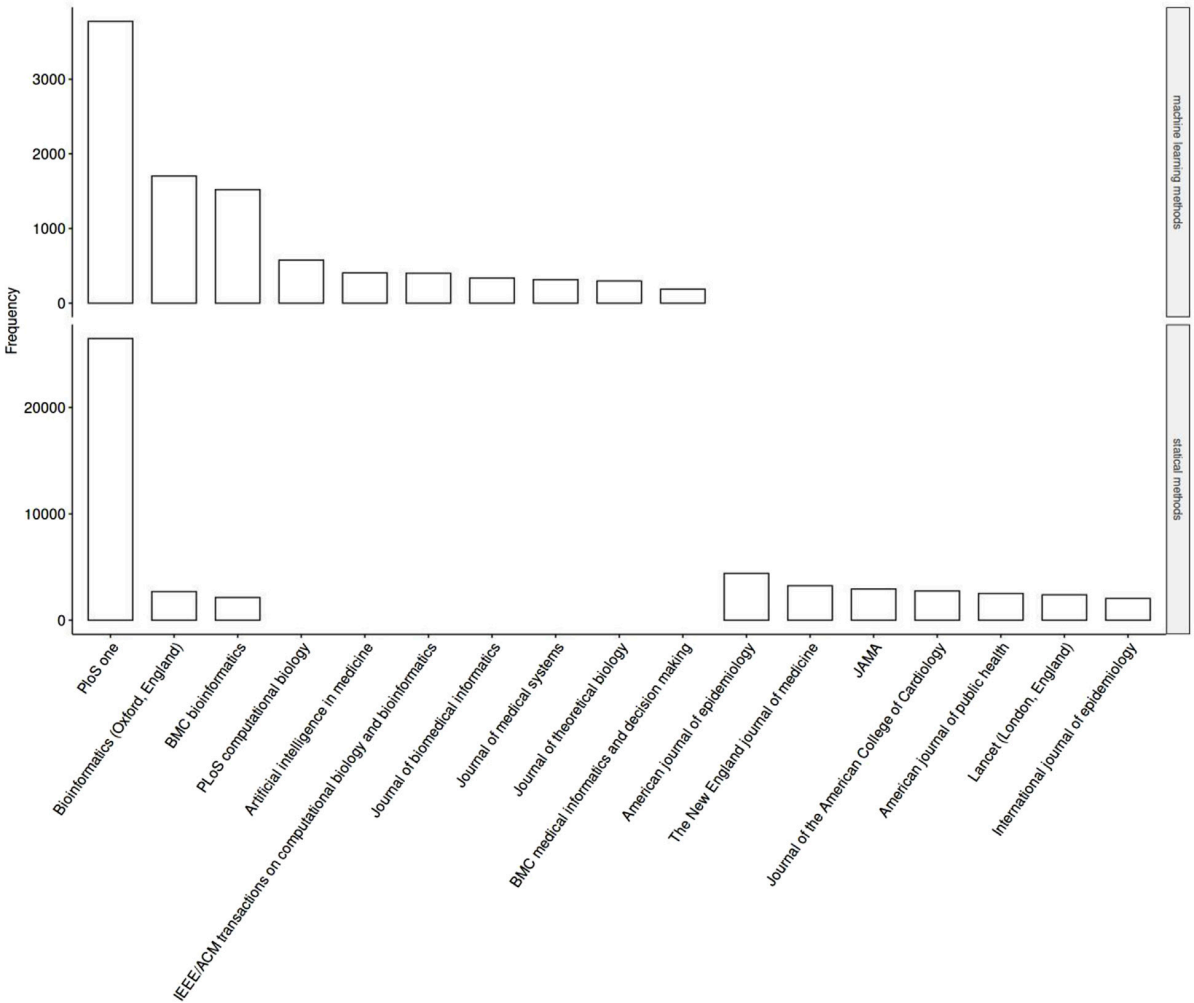
Frequencies of medical subject headings terms for methods associated with machine learning (top chart) and for “classical” statistical methods (bottom chart) (Germany, 2024).

In the first step of our analysis, we investigated whether researchers in epidemiology using statistical methods and researchers using machine learning methods constitute two distinct research communities that publish their work in different journals. We inspected the journals where publications with the MeSH terms associated to *machine learning methods* and *statistical methods* (i.e., the two communities) present their research, finding in fact two distinct groups of journals, with only a slight overlap represented by PLOS One, where research from both communities appear) (Figure 2; see Supplementary Appendix SA.2).

To understand and properly interpret these preliminary findings, we conducted some interviews with bioinformaticians, genome researchers and epidemiologists, questioning them about the relationship between bioinformatics and epidemiology in the medical field. The interviews confirmed a trend towards an increase in the use of big data and machine learning, but at the same time the presence of significant obstacles. Epidemiologists increasingly need the results of bioinformatics, but the two disciplines still seem to be separated in approach, methods, and reference journals.

To investigate further, we reran the analysis using structural topic modeling (STM) [30]. STM is a method in natural language processing that enhances traditional topic modeling by incorporating document metadata, such as publication dates, authors, or journal types, into the analysis. This allows STM to identify latent topics within a corpus and understand how these topics vary with different covariates.

For our analysis, we used the publication date as the covariate (see Supplementary Appendix SA.3). The analysis was conducted using the R package stm (version 1.3.7) with R version 4.3. In topic modeling, it is necessary to fix the total number of topics. Initially, we ran a model comparison with topic numbers ranging from 5 to 150, with a step-size of 10. Based on statistical measures of exclusivity and coherence, we found no clear models with an optimal trade-off between these metrics. Therefore, we qualitatively inspected the models with k values from 50 to 90 and k = 150.

For each model, we labeled each topic and investigated which model provided sufficient resolution for our research question, combining expertise in epidemiology and data science. In doubtful cases, we further examined the abstracts for each topic and classified the topics as either “population epidemiology” or “clinical epidemiology.”

After labeling topics and inspecting abstracts, we concluded that k = 150 provided sufficient resolution for our purposes. We then analyzed the topics over time (see Supplementary Appendix SAB). To investigate our hypothesis, we divided the first 40 topics into three groups: those closer to the clinical side of epidemiology (17 topics), those closer to the population side of epidemiology (15 topics), and those that could not be plausibly assigned to either side (black-labeled topics) (8 topics). See Supplementary Appendix SA.3 for the list of topics.

## Results

**FIGURE 3:**
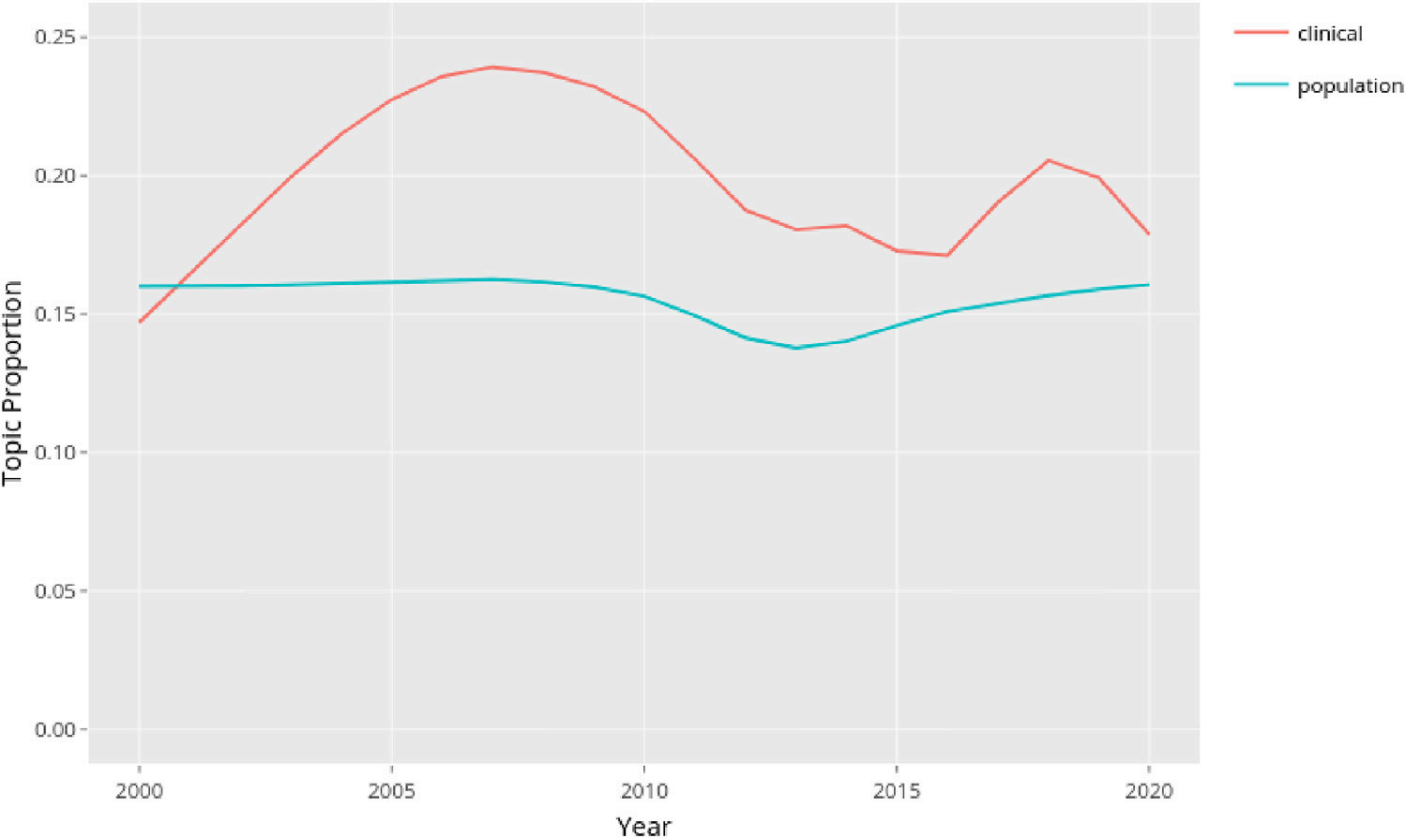
Trend of the topics grouped as closer to the clinical side of epidemiology (17 topics) or to the population side of epidemiology (15 topics) (Germany, 2024).

In the following step we analyzed over time the topics grouped in this way (see Supplementary Appendix SAB). We looked at the topic proportion for each group as indication of the relative importance of different topics within the corpus. The proportion is calculated based on number of words assigned to the topic divided by total number of words for each document in the corpus. Our investigation showed that the prevalence of topics referring to population epidemiology remained quite stable in the 20 years of our investigation, whereas the topics associated with the clinical side of epidemiology had a significant increase between 2000 and 2008, a slight decrease until 2016 and a new increment in the following years (Figure 3).

**FIGURE 4:**
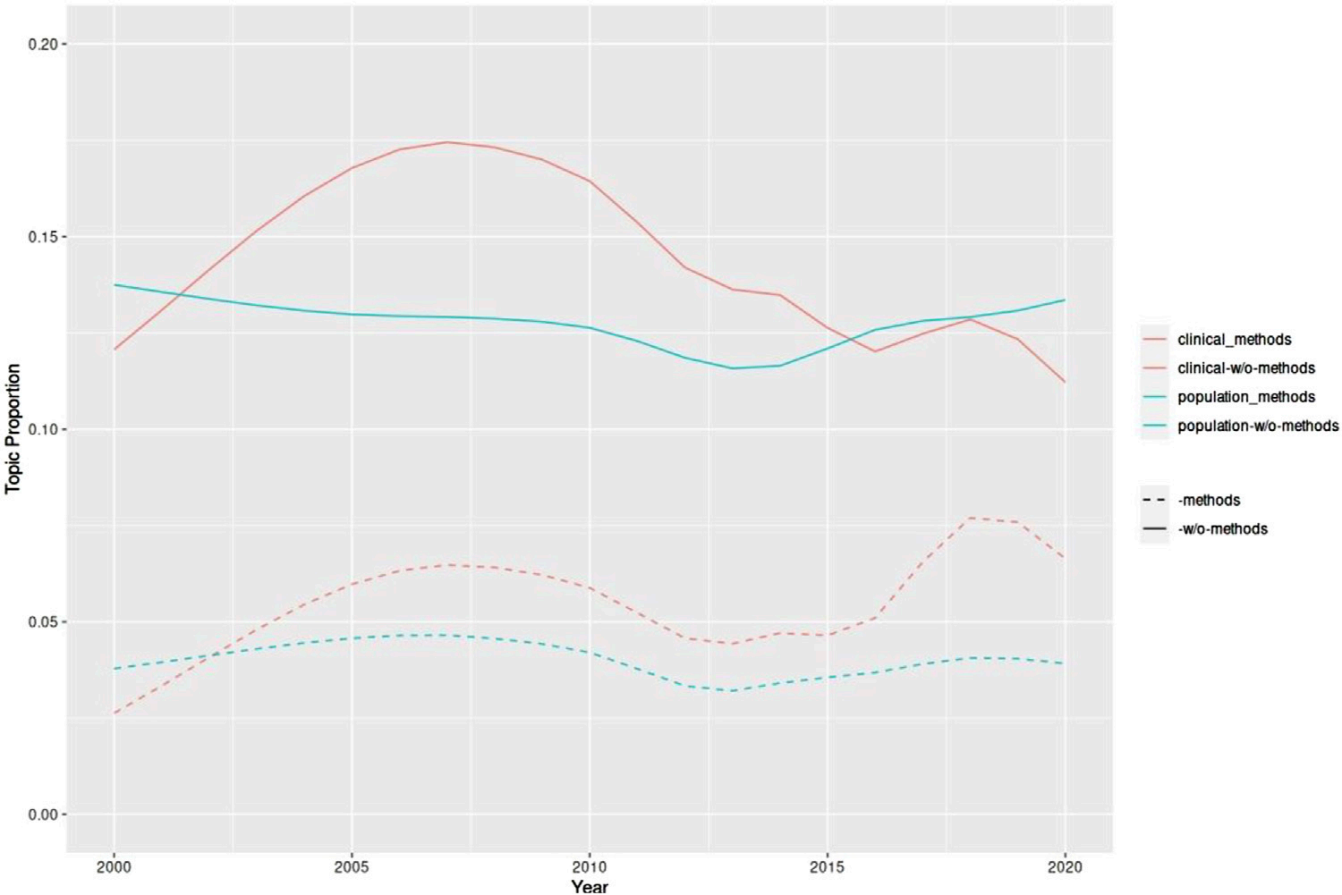
Trend of the topics grouped as closer to the clinical side of epidemiology or to the population side of epidemiology isolating in both groups the topics related to methods (Germany, 2023).

Figure 3 shows that during the period under observation the topics grouped according to clinical labels showed greater variability than the group of topics referring to population epidemiology. We decided to investigate whether and how the use of innovative computational methods in the two branches of epidemiology played a role in these trends. We therefore isolated the topics related to methods in the two groups and observed the resulting trends. Excluding the topics referring to methods in both groups we got Figure 4.

We get four curves in two different colors. Those in blue refer to population epidemiology; those in red to clinical epidemiology. In both colors, the dashed curve corresponds to methods, the solid curve to the remaining topics. The observation of the blue curves shows a substantial stability over time, which mirrors the trend in the non-disaggregated topics (see Figure 3). In the trends in population epidemiology there were apparently no major changes either in the methods or in the other topics over the period we considered.

Conversely, both red curves corresponding to the clinical grouping show greater dynamism over the 20-year observation period. Whereas until 2015 the trend of the two curves is basically parallel, starting in 2016 the curve of method-related topics presents a much more prominent peak. We can assume that this peak in the curve of methods justifies the second peak in the curve of non-disaggregated topics of the clinical side of epidemiology that we have shown in Figure 3. This observation seems to reinforce the idea that in recent times (concomitantly with the development of the latest algorithmic techniques) methodological innovations played a relevant role in clinical epidemiology, whereas in population epidemiology this has not been the case.

This result leads us to suppose that a new separation between clinical epidemiology and population epidemiology is emerging, that runs counter to the trend toward approximation and methodological uniformity in the last decades of 1900. Clinical epidemiology seems to have taken more advantage of recent developments in algorithmic techniques and machine learning, moving closer to bioinformatics, whereas population epidemiology seems to be less inclined to adopt these innovations, or at any rate to do it more slowly.

## Discussion

Our investigation indicates a possible shift in orientation in recent years, showing that the work of epidemiologists changed as a result of the availability of advanced machine learning techniques. The analysis of scientific publications in the epidemiological area suggests that research addressing environmental macro aspects continues to use predominantly statistical methods and has developed in a largely stable manner over the past few decades. In contrast, research with a micro approach addressing the clinical side of the discipline shows a more dynamic trend and an interesting proximity to innovative algorithmic computational methods.

Regarding how the role of epidemiology has shifted relative to other biomedical disciplines and research areas outside the medical field, we observed that the affinity of the clinical approach to algorithmic technologies and bioinformatics research is not surprising. The traditional focus on single patients and their individual needs seems to be accentuated and fostered by algorithmic technologies aimed at the identification of specific clusters of individuals. Far more complex is the case of population epidemiology, which targets macro factors and tries to identify patterns, trends, and outcomes that may be applicable to an entire population. This approach is much more akin to the approach of traditional statistics.

Regarding the impact of computational techniques on epidemiological research, one might conclude that the macro side of epidemiology by its very nature cannot take advantage of this innovation and advances in algorithmic procedures lead toward a renewed methodological separation between the clinical and the ecological aspects of the discipline.

Things are not so simple. On the contrary, some authors argue that precisely these new technologies make it possible (and desirable) to look for a novel connection between molecular modifications at the micro level and the ecology of disease at the macro-environmental level [31].

Other research explores the possibility to directly adopt algorithmic techniques at the community level. The expression “precision public health” (PPH) refers to the attempt to leverage the same technologies that propel PM to “more granularly predict and understand public health risks and customize treatments for more specific and homogeneous subpopulations” [32–34]. Whereas precision medicine is about providing “the right treatment to the right patient at the right time” [35], precision public health aims at providing “the right intervention to the right population at the right time” [36]. PPH could use enhanced epigenetic data and bioinformatic capacity for issues like “precision prevention” [37]. Recent innovative population screening programs, on the other hand, as, for example, the Tempo-Mirai project [38], use the predictive ability of deep learning technologies to personalize screening regimes, advancing early detection while reducing overscreening.

Our work represents a first step towards analyzing the impact of algorithmic procedures on the divergence between population epidemiology and clinical epidemiology. However, our investigation has some limitations.

Firstly, we restricted our analysis to the period before the coronavirus pandemic. During the pandemic, scientific research advanced significantly and adopted innovative predictive techniques [39]. Since our initial research question focused on internal changes in epidemiology related to the advent of algorithmic procedures, we limited the time span to provide a clear picture of this dynamic. We chose to examine publications up until the onset of the COVID-19 pandemic because the pandemic profoundly shifted the focus and volume of medical research, leading to a substantial increase in COVID-19-related studies and potentially sidelining other critical medical topics. Including these pandemic-era publications could skew our literature review, biasing and distorting the pre-pandemic research landscape we aimed to study.

By confining our analysis to the pre-pandemic period, we ensure a more accurate and representative assessment of medical research trends and topics, preserving the integrity and relevance of our findings without the undue influence of the unprecedented global health crisis. Nevertheless, we acknowledge that a follow-up study must include the impact of the pandemic and the post-pandemic era to provide a comprehensive understanding of the ongoing changes in the field. A second limitation is that we only used structural topic modeling for the quantitative analysis. Future work should also include neural-based techniques, such as top2vec [40], which can also give a distance metric between topics.

For the field of epidemiology, technological innovation related to algorithmic techniques represents a significant challenge and potentially a great opportunity. To face the complexity of recent developments, the discipline needs to question its development and the articulations produced inside epidemiology - not least to avoid passively following technological evolution toward a drift toward further internal separation and obstacles to collaboration.
