# Supplementary material for: Precision Epidemiology: A Computational Analysis of the Impact of Algorithmic Prediction on the Relationship Between Population Epidemiology and Clinical Epidemiology
Source: Int J Public Health. 2024 Oct 1;69:1607396. doi: 10.3389/ijph.2024.1607396 (PMC11473335; doi:10.3389/ijph.2024.1607396)
Supplement: Supplementary file 1 [file DataSheet1.pdf]

Appendix A

A.1 Data acquisition

We queried PubMed for research articles on epidemiology using the mesh terms (https://meshb.nlm.nih.gov/search) for *epidemiology* and *epidemiological methods* (see A.1.1.1 for the query string). To distinguish between different methods used in epidemiology, we queried for methods associated with machine learning (see A.1.1.2) or "classical" statistical methods (see A.1.1.3). We used the query string for machine learning based on previous work that investigated machine learning methods in clinical prediction (Christodoulou et al. 2019). The queries on statistical methods were selected based on our team expertise on Mesh terms associated with statistical approaches.

The query results exceeded 1 million publications; we decided to restrict our analysis to the most important journals. We identified the top journals on medicine and health, epidemiology, computational biology, and medical informatics using Google scholar (https://scholar.google.com/citations?view_op=top_venues&hl=en; see A.1.1.4).

The queries resulted in 10822 publications for machine learning methods, 66408 publications for statistical methods (5706 overlapping publications). We have collected N=67838 publications in total, including abstracts, authors, keywords, departments, and year of publication for further inspection. Figure S1 shows the publication count per year for both data set combined.

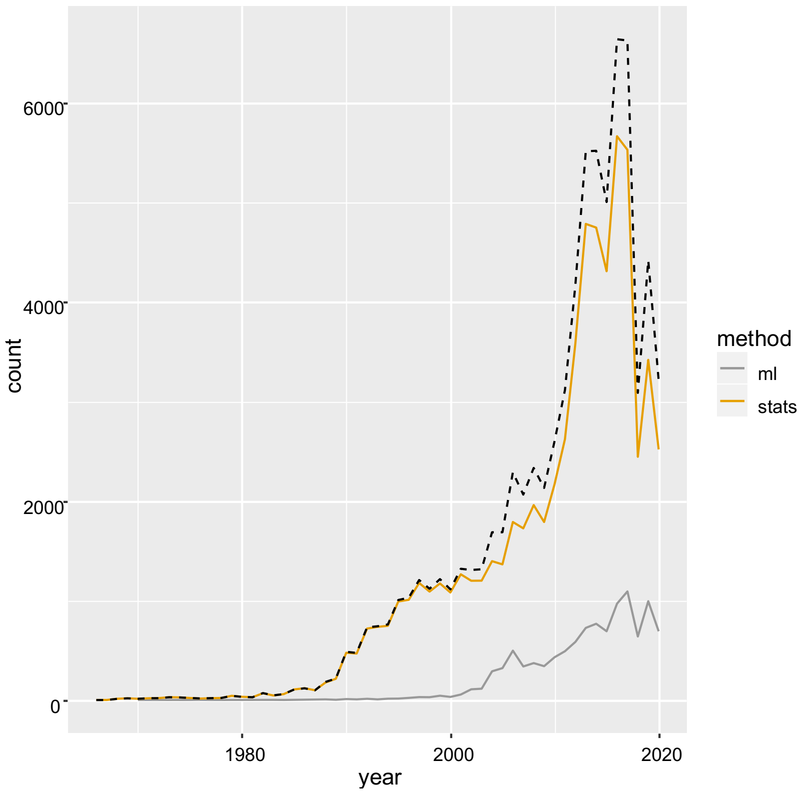

Figure S1: Publications per year for machine learning (ml) and statistical methods (stats) corpus. Dashed line indicates combined number of publications (Bielefeld, Germany, 2023).

A.1.1 Query Strings

A.1.1.1 Epidemiology

epidemiological methods[mesh] OR epidemiology[mesh]

A.1.1.2 Machine Learning

"Machine Learning"[Mesh] or OR support vector machine[MeSH Major Topic] OR neural networks[MeSH Major Topic] OR support vector machine OR multilayer perceptron OR neural network OR random forest OR lasso OR ridge OR kernel OR bayesian network OR classification tree OR regression tree OR relevance vector machine OR nearest neighbor OR probability estimation tree OR elastic net OR ensemble OR penalized OR regularized OR bagging
OR boosting OR fuzzy OR Naive bayes OR deep learning OR genetic algorithms

A.1.1.3 Statistical Methods

"Survival Analysis"[Mesh] or "Factor Analysis, Statistical"[Mesh] or "Biostatistics"[Mesh] or "Discriminant Analysis"[Mesh] or "Models, Statistical"[Mesh] or "Regression Analysis"[Mesh] or "Analysis of Variance"[Mesh] OR "Cluster Analysis"[Mesh] OR "Statistics, Nonparametric"[Mesh] OR "Data Interpretation, Statistical"[Mesh] or logistic models[MeSH Terms] OR multinomial logistic regression OR ordinal logistic regression OR logistic regression OR proportional odds regression

A.1.1.4 Journals

"The New England Journal of Medicine"[Journal] OR "JAMA"[journal]OR "lancet"[journal] or "PLoS ONE"[journal] or "Nature Genetics"[journal] or "Nature Medicine"[journal] or "The Lancet Oncology"[journal] or " American journal of epidemiology "[journal] or " International journal of epidemiology "[journal] or "Epidemiology"[journal] or " Journal of clinical epidemiology" [journal] or " Annals of epidemiology"[journal] or " British medical journal"[journal] or " American journal of public health" [journal] or "Cell"[journal] or "Emerging Infectious Diseases"[journal] or "Journal of Epidemiology and Community Health"[journal] or "European Journal of Epidemiology"[journal] or "International Journal of Hygiene and Environmental Health"[journal] or \Bioinformatics"[journal] or \PLOS Computational Biology"[journal] or \BMC Bioinformatics"[journal] or \Briefings in Bioinformatics\[journal] or \GigaScience"[journal] or \Database: The Journal of Biological

Databases and Curation"[journal] or \Journal of Theoretical Biology"[journal] or \IEEE/ACM Transactions on Computational Biology and Bioinformatics"[journal] or \Genomics, Proteomics & Bioinformatics"[journal] or \BMC Systems Biology" [journal] or \Journal of Mathematical Biology"[journal] or \Mathematical Biosciences"[journal] or \Journal of Medical Internet Research"[journal] or \Journal of the American Medical Informatics Association"[journal] or \Journal of Biomedical Informatics"[journal] or "International Journal of Medical Informatics"[journal] or "Journal of Medical Systems"[journal] or "BMC Medical Informatics and Decision Making"[journal] or "Artificial Intelligence in Medicine"[journal] or "Journal of Telemedicine and Telecare"[journal]

A.2 Keyword Inspection

As a first analysis, we inspected the distribution of keywords regarding the publication development over the last years and the journals where the two communities publish their research.

A.2.1 Journals

First, we wanted to investigate whether there might be two distinct research communities that present their work in different venues. We calculated the frequencies of publications per journal according to research focussing on ML or statistical methods and selected the most extensive top 10 journals. Figure 2 in the main text shows a bar plot with the frequencies of publications for each journal. As the plot shows, there is mainly one journal in which researchers from both corpora publish: *PloS ONE* (https://journals.plos.org/plosone/), which is a peculiar online-only peer-reviewed scientific journal, published by the Public Library of Science (PLOS) since 2006 and covering primary research from any discipline within science and medicine. It is different from traditional scientific publishing in that it only verifies whether experiments and data analysis were conducted rigorously, and leaves it to the scientific community to ascertain importance, post publication, through debate and comment.

The distribution according to journals shows a quite distinct pattern. Research using statistical methods in epidemiology is published in the *American journal of epidemiology, The new england journal of medicine, jama, journal of the american college of cardiology, lancet, and the internation journal of epidemioloy*. Research using methods from ml is published in *AI in medicine, transactions on computational biology and bioinformatics, journal of medical systems, journal of theoretical biology, BMC medical informatics and decision making*. There is a slight overlap between the corpora. Besides *PloS ONE* as a common venue, both corpora include publications that were published in *Bioinformatics,* and *BMC Bioinformatics*.

A.2.2 Keywords

Finally, we investigated the keywords associated with the publications. Each publication is tagged with multiple mesh terms and keywords. We removed from the further analysis keywords that are frequent but non informative for our research question. These keywords are: *'Adult'*, *'Animals'*, *'Female'*, *'Male'*, *'Middle Aged'*, *'Adolescent'*, *'Aged'*, *'Aged, 80 and over'*, *'Young Adult'*, *'Humans'*, *'Child'* , *'United States'*.

Figure S2 and Figure S3 show the relative frequencies for each keyword in both corpora. Each frequency count is normalized by the total number of publications per year. It shows the ratio of publications that are tagged with the keyword. This number does not sum up to one since publications can have multiple keyword tags.

As we can see in Figure S2, there is a peak for Algorithms and Statistical models around 2005 for the statistical method data set. From 2005 on, the usage of these keywords dropped significantly. A similar drop can be found in the machine learning corpus. The keyword Algorithm dropped from .8 in 2005 to .42 in 2019.

Only three of the top 20 keywords can be found in both data sets: Algorithms, Statistical Models, and Cluster Analysis (see Figure S2 and S3)

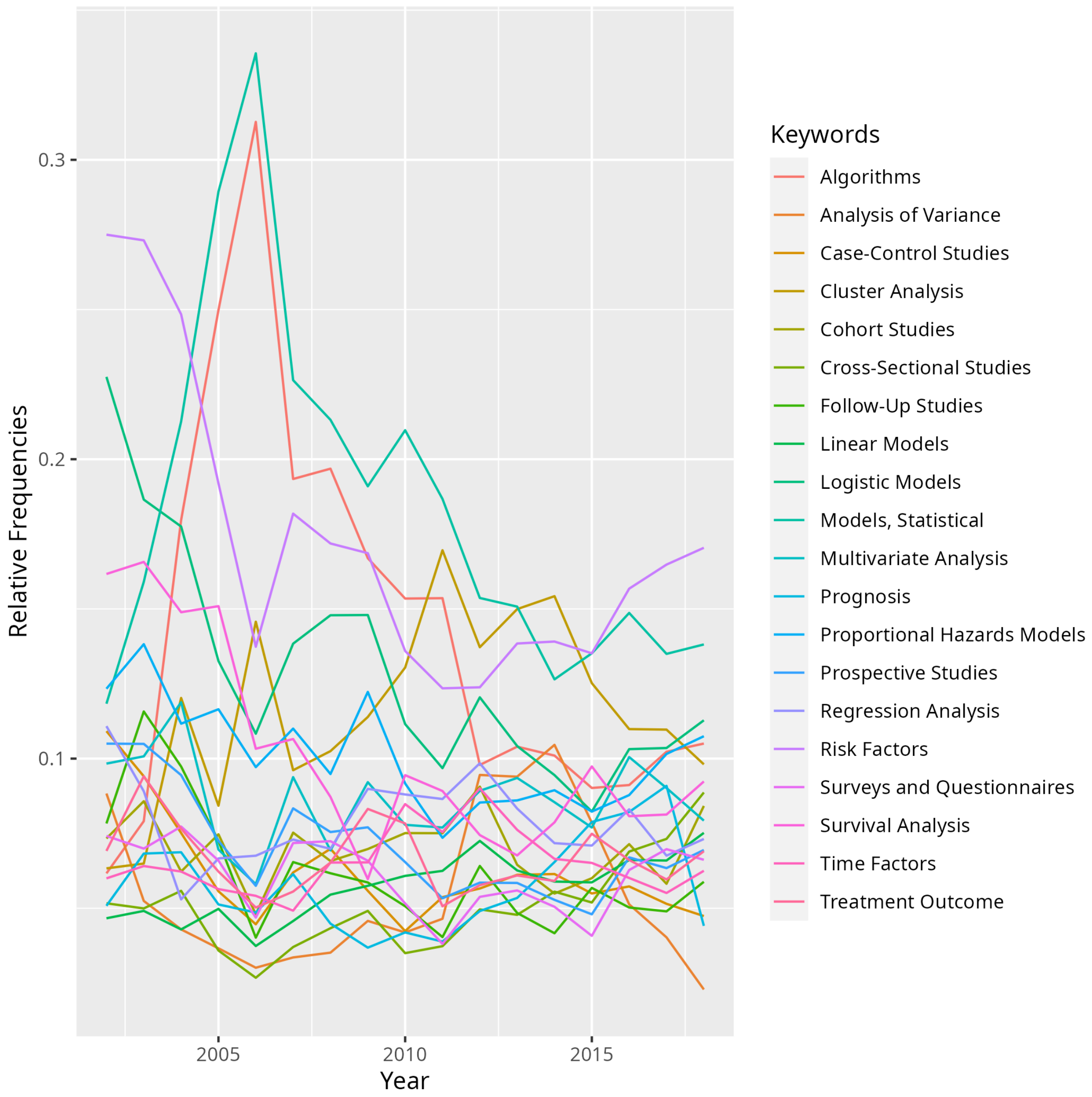

Figure S2: Relative keyword counts normalized by the total number of publications per year for the statical method data set (Bielefeld, Germany, 2023).

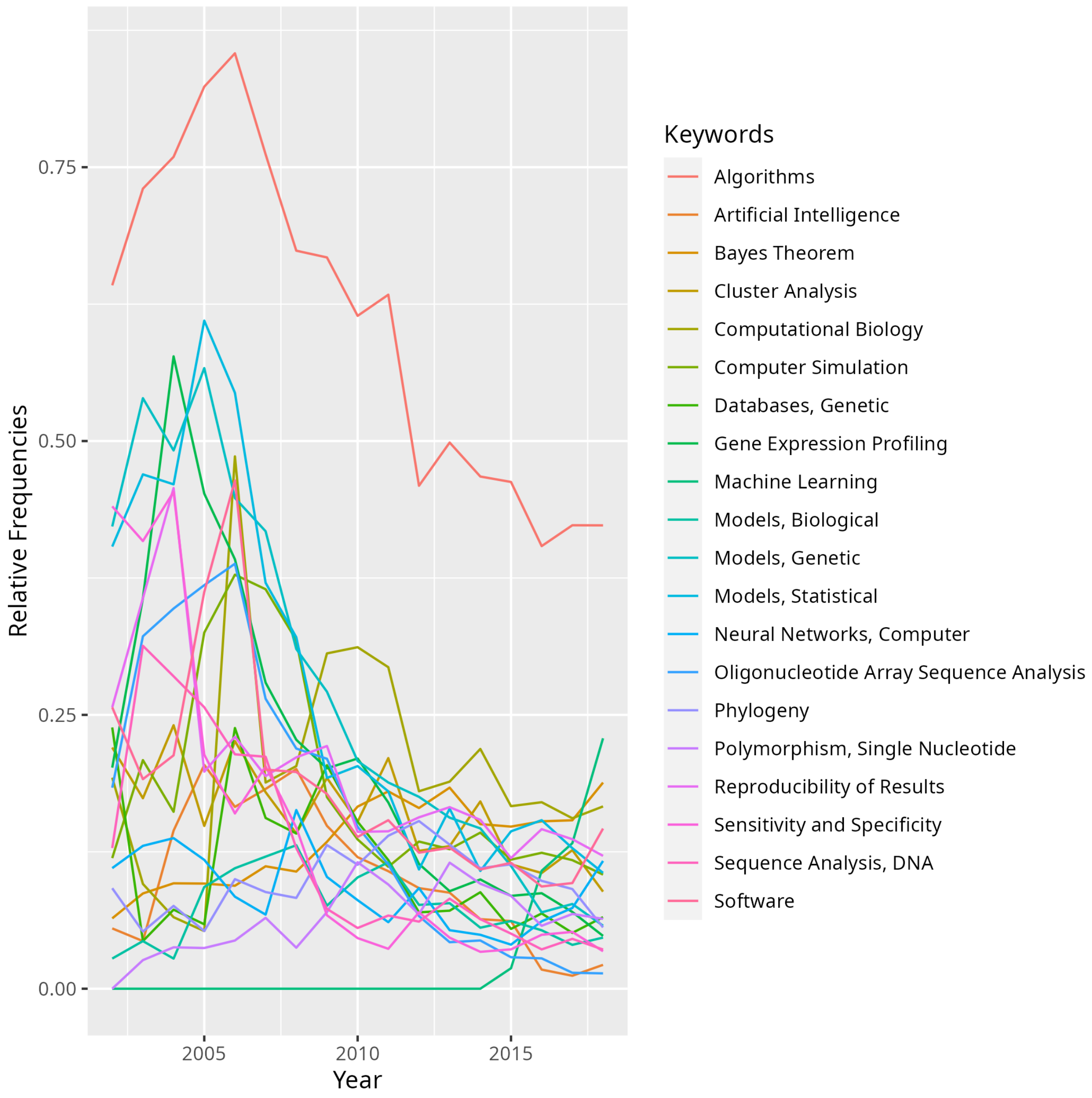

Figure S3: Relative keyword counts normalized by the total number of publications per year for the machine learning data set (Bielefeld, Germany, 2023).

Figures S4, S5 and S6 show a comparison of the relative frequencies of the three keywords that can be found in both data sets: Algorithms, Statistical Models, and Cluster Analysis. Figure S4 presents evidence that the usage of the keyword *statistical models* significantly dropped over the past two decades. From usage as a keyword in 60 percent of the publications in 2005 it dropped below 20 percent per year after 2010.

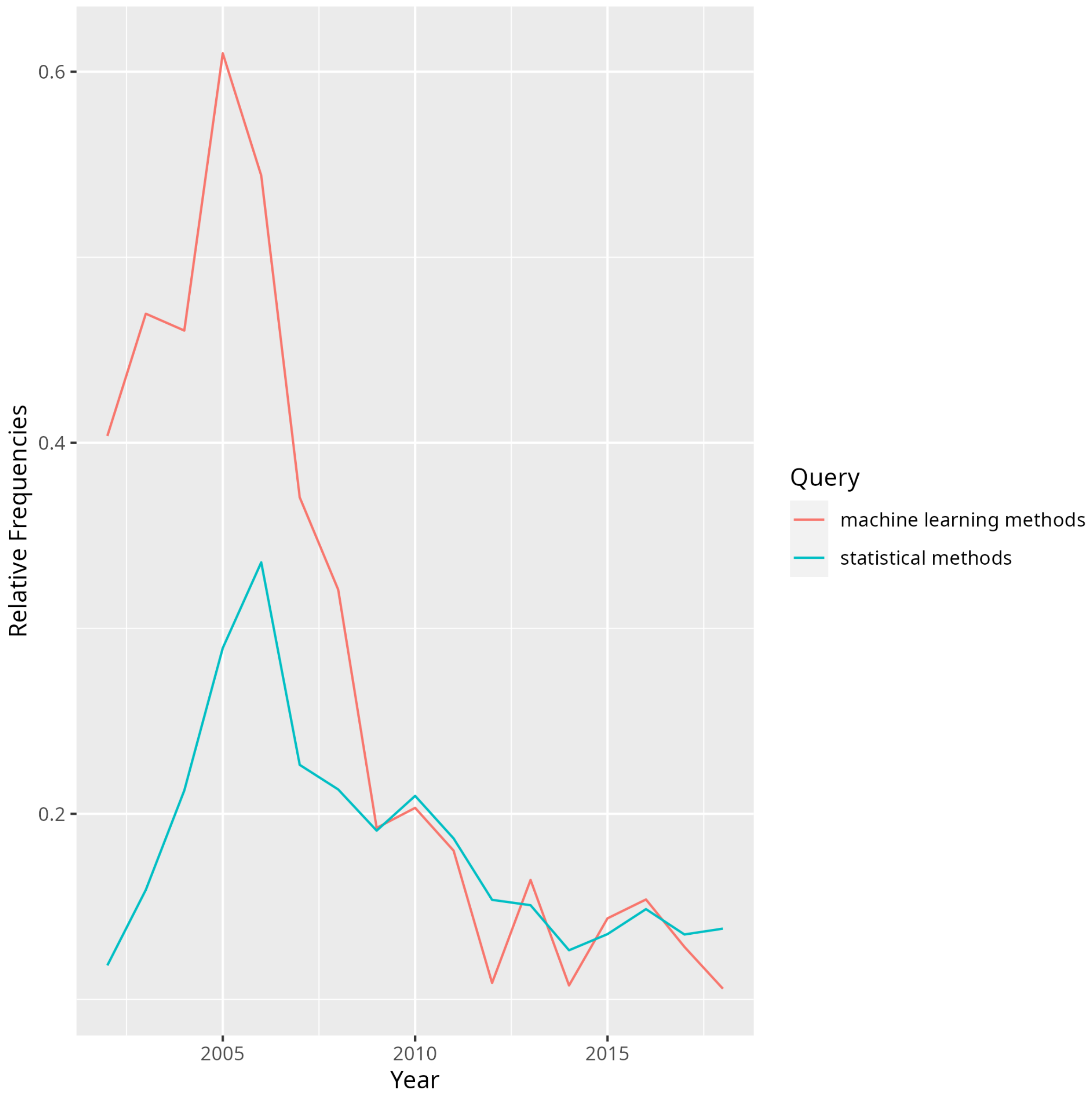

Figure S4: Relative frequencies for the keyword Statistical models (ML red line; Stats blue line) (Bielefeld, Germany, 2024).

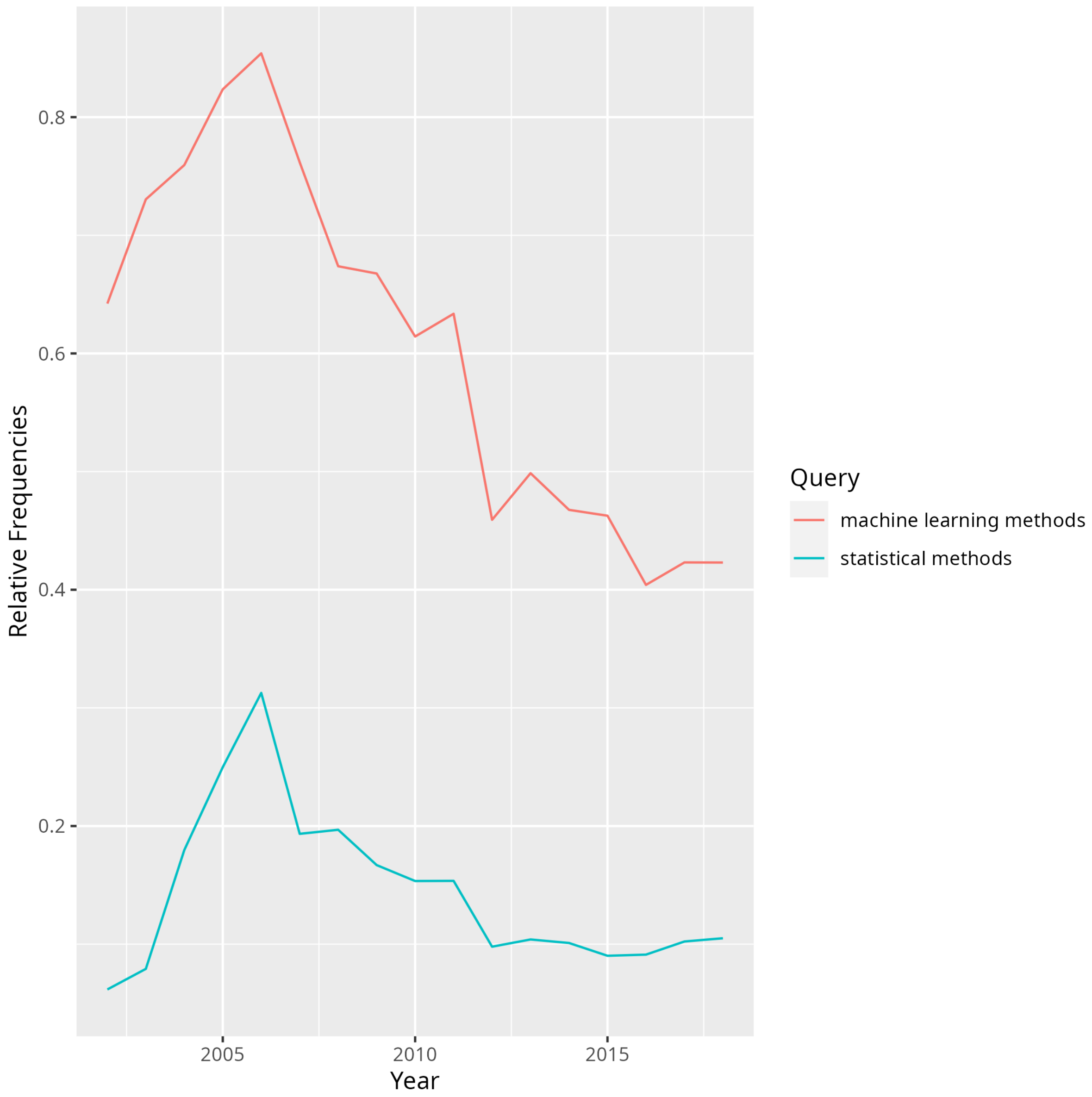

Figure S5: Relative frequencies for the keyword Algorithms (ML red line; Stats blue line) (Bielefeld, Germany, 2024).

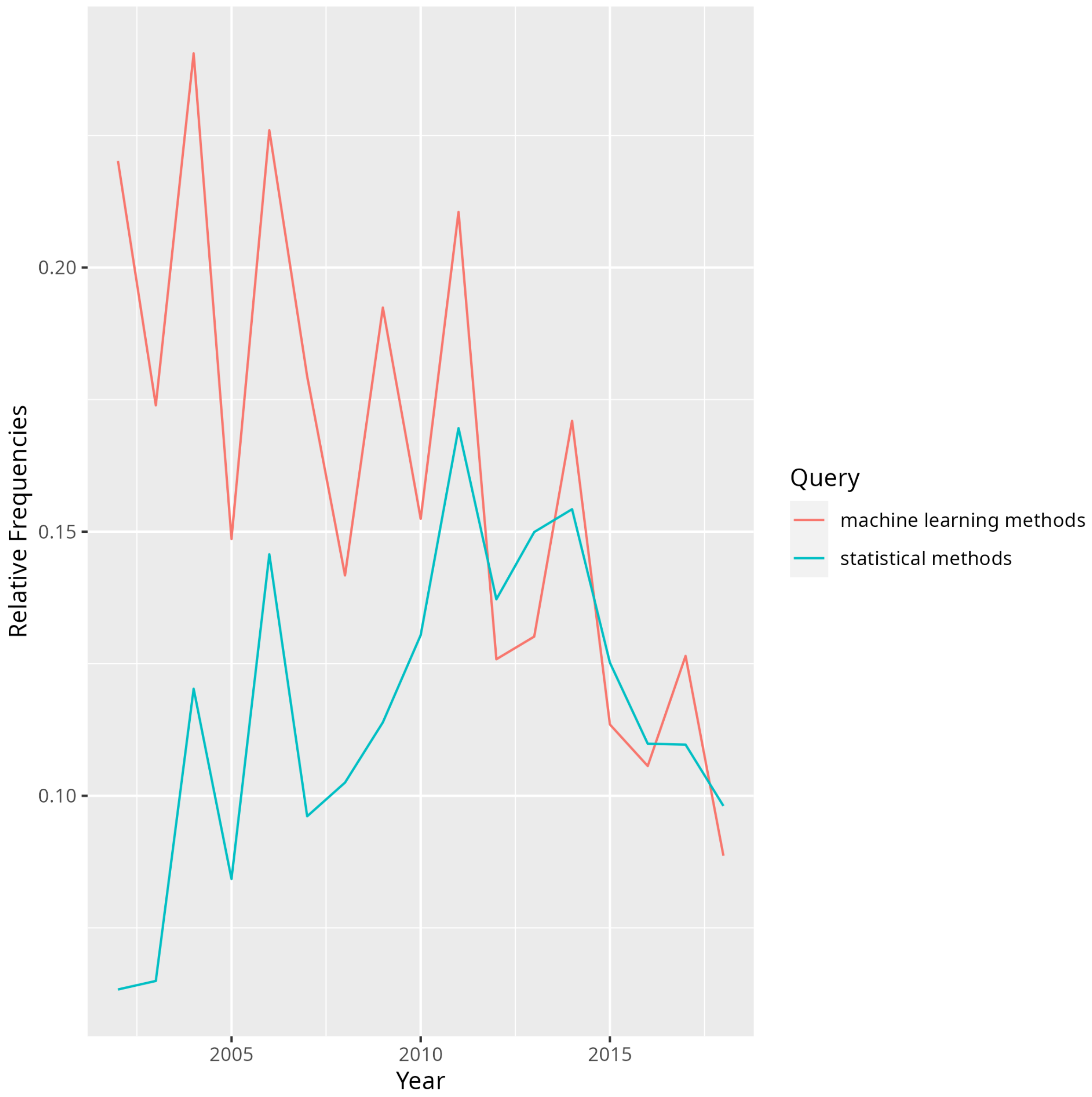

Figure S6: Relative frequencies for the keyword Cluster Analysis (ML red line; Stats blue line) (Bielefeld, Germany, 2024).

A.3 Structural Topic Modeling

To investigate latent research trends in the field of epidemiology, we quantitatively mapped the scientific discourse according to research trends, topics and communities using Structural Topic Modelling, which includes document level covariate information (e.g., publication year). Covariates can improve inference and qualitative interpretability and affect topical prevalence, content or both. We used the R package named stm. We had to

decide the number of topics before the modelling step. This time we expected that relying solely on statistical measures could lead to a suboptimal model parameter choice. We first ran a model comparison with topic numbers ranging from 5 to 150 with a step-size of 10. Based on statistical measures using exclusivity and coherence we found that there are no clear models with an optimal trade-off between exclusivity and coherence. Thus, we additionally qualitatively inspected the models from k=50 until k=90 and k=150. For each model, we labelled each topic and investigated which model gave us a fine enough resolution to pursue our research question. We further investigated the abstracts for each topic and classified the topics belonging either to the group of 'population epidemiology' or to 'clinical epidemiology'.

After labelling topics and inspecting abstracts, we concluded that k=150 gives us a sufficient resolution for our purposes and analysed the topics over time (see Appendix B).

Below is the list of topics we considered. In our analysis, we decided to manually delete the topic 109 “Economic market” and replace it with the topic 16 “Vector borne disease".

Highest Prob are the words within each topic with the highest probability
FREX are the words that are both frequent and exclusive, identifying words that distinguish topics.

Blue: Topics related to population epidemiology
Green: Topics related to clinical epidemiology
Black: Generic topics not plausibly related to one or the other sides of epidemiology

k=150

Topic 111 Top Words: risk factor analysis (logistic regression)

Highest Prob: risk, factor, associ, case, control, regress, logist

FREX: factor, risk, logist, odd, case, regress, control

Topic 55 Top Words: effect size estimation/selection bias

Highest Prob: estim, effect, bias, error, measur, result, correct

FREX: bias, error, causal, assumpt, estim, true, correct

Topic 89 Top Words: bayesian methods/simulation

Highest Prob: comput, algorithm, simul, implement, effici, bayesian, propos

FREX: packag, softwar, bayesian, supplementari, mont, carlo, framework

Topic 117 Top Words: statistics

Highest Prob: variabl, statist, sampl, distribut, size, regress, correl

FREX: variabl, miss, varianc, imput, power, distribut, statist

Topic 147 Top Words: classification

Highest Prob: featur, classif, classifi, learn, perform, propos, select

FREX: machin, classif, svm, featur, learn, classifi, kernel

Topic 28 Top Words: stochastic mathematical models

Highest Prob: model, paramet, fit, linear, equat, develop, dynam

FREX: model, mathemat, equat, fit, paramet, stochast, nonlinear

Topic 29 Top Words: bioinformatics and genome research

Highest Prob: gene, cluster, express, set, biolog, analysi, dataset

FREX: cluster, dataset, gene, microarray, biolog, biclust, hierarch

Topic 116 Top Words: text mining

Highest Prob: system, inform, research, base, knowledg, user, provid

FREX: text, mine, biomed, expert, ehr, fuzzi, topic

Topic 114 Top Words: prediction

Highest Prob: predict, valid, sensit, specif, curv, perform, clinic

FREX: curv, predict, auc, sensit, valid, diagnost, roc

Topic 112 Top Words: trends

Highest Prob: rate, increas, chang, state, trend, decreas, period

FREX: rate, trend, declin, state, rise, annual, unit

Topic 13 Top Words: ecological topology

Highest Prob: network, biolog, connect, system, dynam, structur, complex

FREX: network, graph, topolog, connect, modular, edg, boolean

Topic 145 Top Words: health survey

Highest Prob: health, survey, inform, public, servic, access, report

FREX: health, internet, caregiv, public, servic, access, messag

Topic 46 Top Words: gene expression

Highest Prob: gene, express, pathway, regul, transcript, identifi, analysi

FREX: pathway, transcriptom, transcript, regul, gene, express, enrich

Topic 60 Top Words: sequence alignment

Highest Prob: sequenc, align, rna, base, read, similar, structur

FREX: align, read, sequenc, assembl, hmm, rna, pipelin

Topic 51 Top Words: clinical trials

Highest Prob: intervent, report, trial, clinic, review, recommend, design

FREX: guidelin, intervent, recommend, journal, review, complianc, citat

Topic 96 Top Words: cox regression

Highest Prob: ratio, hazard, adjust, interv, confid, associ, proport

FREX: hazard, rr, cox, ratio, proport, adjust, confid

Topic 71 Top Words: risk factor: age

Highest Prob: age, year, life, earli, associ, function, cognit

FREX: life, longitudin, dementia, cognit, elder, telomer, frailti

Topic 68 Top Words: cognitive visual tasks

Highest Prob: task, process, visual, stimulus, respons, neural, neuron

FREX: stimulus, auditori, eeg, spike, task, sound, paradigm

Topic 38 Top Words: prevalences

Highest Prob: preval, section, cross, aor, survey, sampl, logist

FREX: preval, section, aor, cross, china, chines, awar

Topic 10 Top Words: risk factor age

Highest Prob: death, year, age, risk, follow, person, relat

FREX: death, excess, registri, die, person, survivor, sweden

Topic 136 Top Words: graph problems

Highest Prob: tree, distanc, maximum, problem, matrix, likelihood, solut

FREX: tree, distanc, solut, partit, reconstruct, maximum, matrix

Topic 9 Top Words: polymorphism

Highest Prob: associ, genotyp, variant, polymorph, allel, snps, gene

FREX: snps, snp, allel, polymorph, variant, genotyp, aa

Topic 74 Top Words: socio economic status

Highest Prob: status, educ, incom, socioeconom, associ, social, disabl

FREX: incom, inequ, socioeconom, educ, marit, unemploy, sis

Topic 44 Top Words: habitats & ecosystems

Highest Prob: speci, spatial, habitat, area, distribut, fore, scale

FREX: habitat, land, landscap, ecosystem, fore, biodivers, fisheri

Topic 90 Top Words: randomized clinical trials

Highest Prob: random, placebo, receiv, trial, dose, primari, mg

FREX: placebo, doubl, blind, assign, random, end, clinicaltrials.gov

Topic 138 Top Words: scale validity

Highest Prob: measur, scale, reliabl, assess, item, valid, correl

FREX: item, instrument, scale, reliabl, agreement, psychometr, exploratori

Topic 88 Top Words: meta-analysis

Highest Prob: analysi, meta, heterogen, effect, pool, search, systemat

FREX: meta, pool, heterogen, systemat, search, embas, summari

Topic 100 Top Words: social psychology/social determination theory

Highest Prob: social, decis, support, person, make, particip, prefer

FREX: decis, belief, prefer, cope, motiv, emot, choic

Topic 3 Top Words: control group comparison

Highest Prob: group, control, differ, signific, compar, similar, comparison

FREX: group, differ, control, divid, comparison, similar, compar

Topic 92 Top Words: protein-protein interaction

Highest Prob: protein, interact, function, predict, domain, complex, residu

FREX: ppi, protein, interact, residu, amino, domain, subcellular

Topic 109 Top Words: economic markets

Highest Prob: polici, market, econom, price, law, countri, trade

FREX: market, price, stock, trade, law, tax, financi

Topic 146 Top Words: social environment

Highest Prob: area, resid, level, individu, urban, neighborhood, citi

FREX: neighborhood, resid, urban, neighbourhood, census, multilevel, depriv

Topic 23 Top Words: treatment response

Highest Prob: treatment, respons, treat, therapi, week, effect, dose

FREX: treatment, treat, respons, week, therapi, untreat, interferon

Topic 7 Top Words: sensitivity/specifity

Highest Prob: test, posit, negat, detect, result, fals, laboratori

FREX: posit, test, negat, fals, laboratori, detect, fisher

Topic 30 Top Words: biological sex

Highest Prob: woman, man, age, year, associ, report, adjust

FREX: man, woman, menopaus, menarch, women, postmenopaus, age

Topic 37 Top Words: peptide structure

Highest Prob: structur, peptid, experiment, molecul, reaction, dynam, conform

FREX: conform, kinet, loop, dock, structur, atom, thermodynam

Topic 64 Top Words: care management

Highest Prob: care, manag, servic, primari, qualiti, nurs, improv

FREX: care, nurs, facil, manag, referr, staff, servic

Topic 78 Top Words: working hours

Highest Prob: time, work, seri, hour, day, shift, monitor

FREX: time, seri, spend, sit, shift, work, mobil

Topic 144 Top Words: genetic phenotypes

Highest Prob: genet, phenotyp, trait, map, variat, individu, complex

FREX: phenotyp, qtl, trait, genet, herit, ld, linkag

Topic 36 Top Words: prognosis survival rate cancer stages

Highest Prob: surviv, stage, ii, iii, prognosi, free, earli

FREX: stage, iii, surviv, iv, ii, kaplan, prognosi

Appendix B

B.1 Topic assignment

Clinical side of epidemiology	Population side of epidemiology	Black-labelled topics
• topic 89: Bayesian methods/simulation (stats/ml method)<br>• topic 147: classification (ml method)<br>• topic 29: bioinformatics and genome research<br>• topic 116: text mining (ml method)<br>• topic 46: gene expression<br>• topic 60: sequence alignment<br>• topic 51: clinical trials<br>• topic 9: polymorphism<br>• topic 90: rct<br>• topic 138: scale validity<br>• topic 3: control group comparison<br>• topic 92: ppi<br>• topic 23: treatment response<br>• topic 30: biological sex<br>• topic 37: peptide structure<br>• topic 64: care management<br>• topic 144: genetic phenotypes	• topic 111: risk factor analysis<br>• topic 55: effect size estimation<br>• topic 117: statistics (stats method)<br>• topic 28: stochastic mathematical models (stats method)<br>• topic 13: ecological topology<br>• topic 145: health survey<br>• topic 71: risk factor: cognitive decline<br>• topic 10: risk factor: age<br>• topic 74: socio economic status<br>• topic 44: habitats & ecosystems<br>• topic 16: vector borne disease surveillance and control<br>• topic 146: social environment<br>• topic 7: sensitivity/specificity<br>• topic 78: working hours<br>• topic 36: prognosis survival rate cancer stages	• topic 114: prediction<br>• topic 112: trends<br>• topic 96: Cox regression<br>• topic 68: cognitive visual tasks<br>• topic 38: prevalence<br>• topic 136: graph problems<br>• topic 88: meta-analysis<br>• topic 100: social psychology theory

B.2 Evolution of Topics proportion

The following figures show the evolution of topic proportions. The topic proportion, or expected topic proportion, is the proportion of documents in our corpus that are expected to belong to a given topic, based on our model's estimated topic proportions. B.1 shows the development of topics associated with clinical epidemiology between the years 2000 and 2001. B.2 presents the same plot for topics related to population epidemiology.

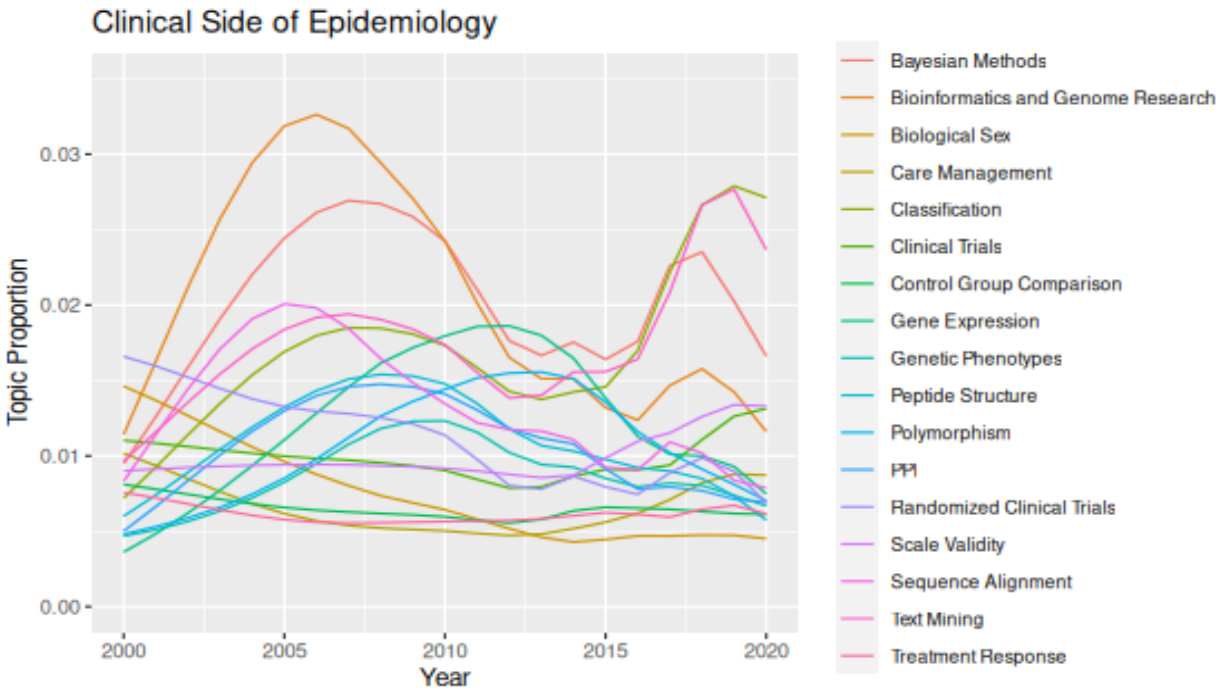

Figure B.1: Temporal development of topic proportions related to the clinical side of epidemiology between 2000 and 2020 (Bielefeld, Germany, 2024).

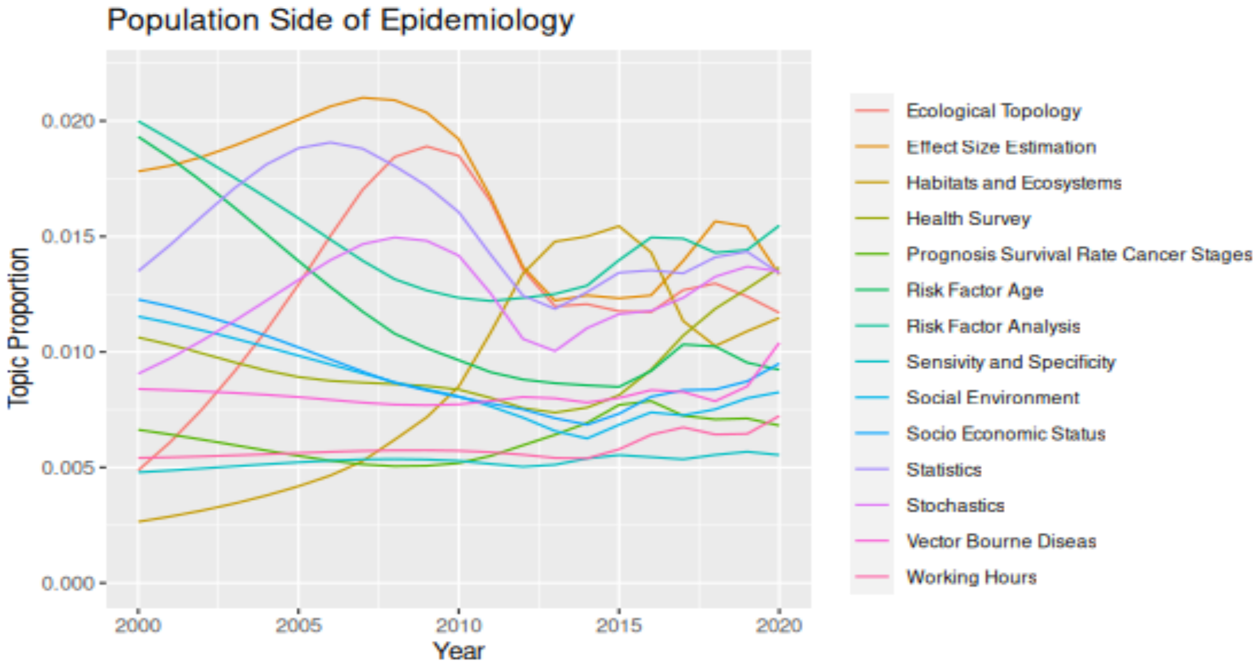

Figure B.2: Temporal development of topic proportions related to the population side of epidemiology between 2000 and 2020 (Bielefeld, Germany, 2024).

We have grouped the topics represented in Figg. B.1 and B.2 and aggregated the topic proportion to get an overview on the development of clinical and population epidemiology between the years 2000 and 2020

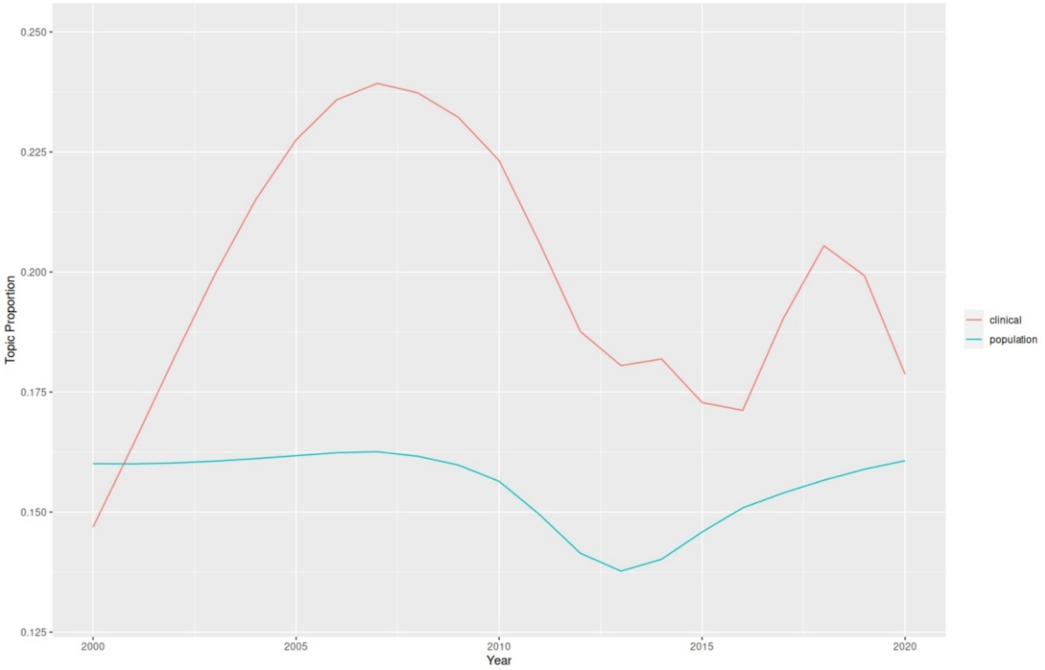

Figure B.3: Summed topic proportion for clinical and population-based epidemiology between 2000 and 2020 (Bielefeld, Germany, 2024).

B.3 Prevalence of clinical and population epidemiology without methods

To understand how and whether methods are used differently in clinical and population epidemiology, we have excluded topics associated to methods for each field of epidemiology. Figures B.4 and B.5 shows the development of topics related to clinical and population-based epidemiology without plotting topics related to methods.

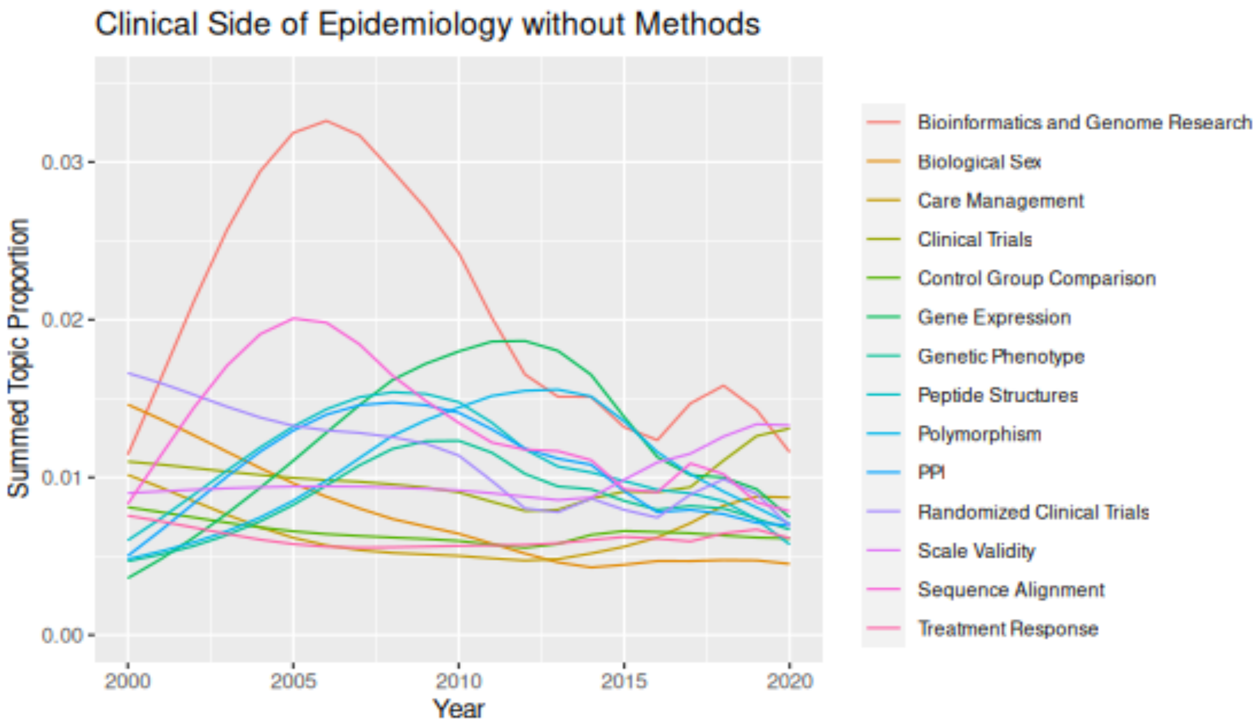

Error! Reference source not found.

Figure B.4: Temporal development of topic proportions related to the clinical side of epidemiology without topics on methods between 2000 and 2020 (Bielefeld, Germany, 2024).

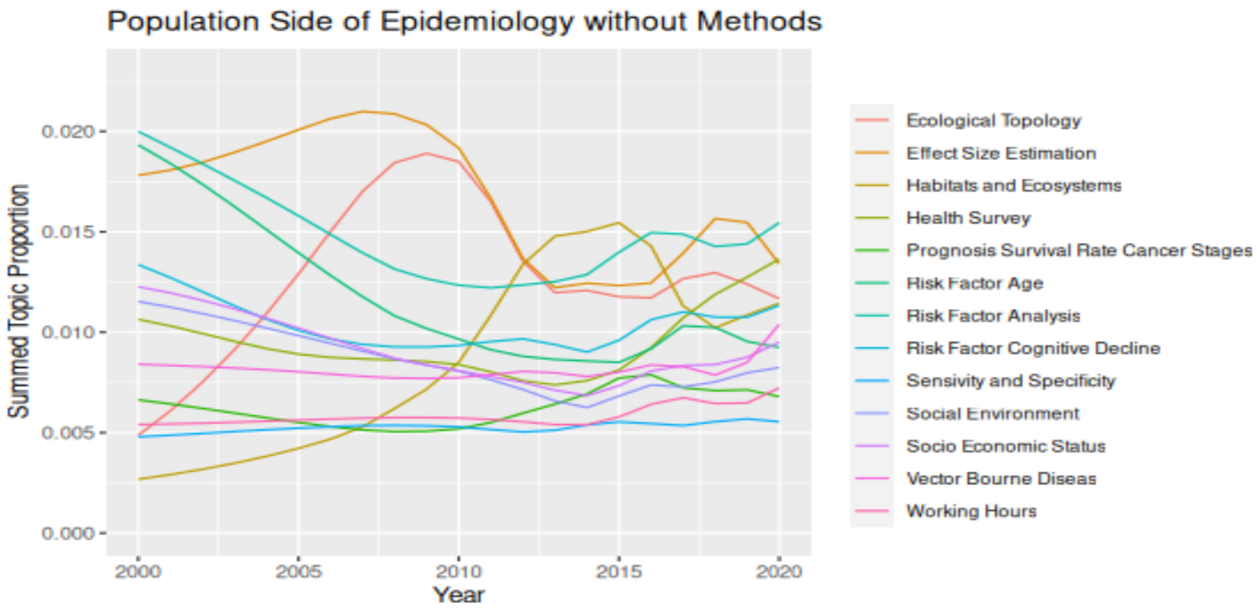

Figure B.5: Temporal development of topic proportions related to the population side of epidemiology without topics on methods between 2000 and 2020 (Bielefeld, Germany, 2024).

Figure B.6 shows the development of aggregated topics for clinical and population epidemiology in total and in comparison, with the resulting trends from isolating the topics related to methods in the two groups.

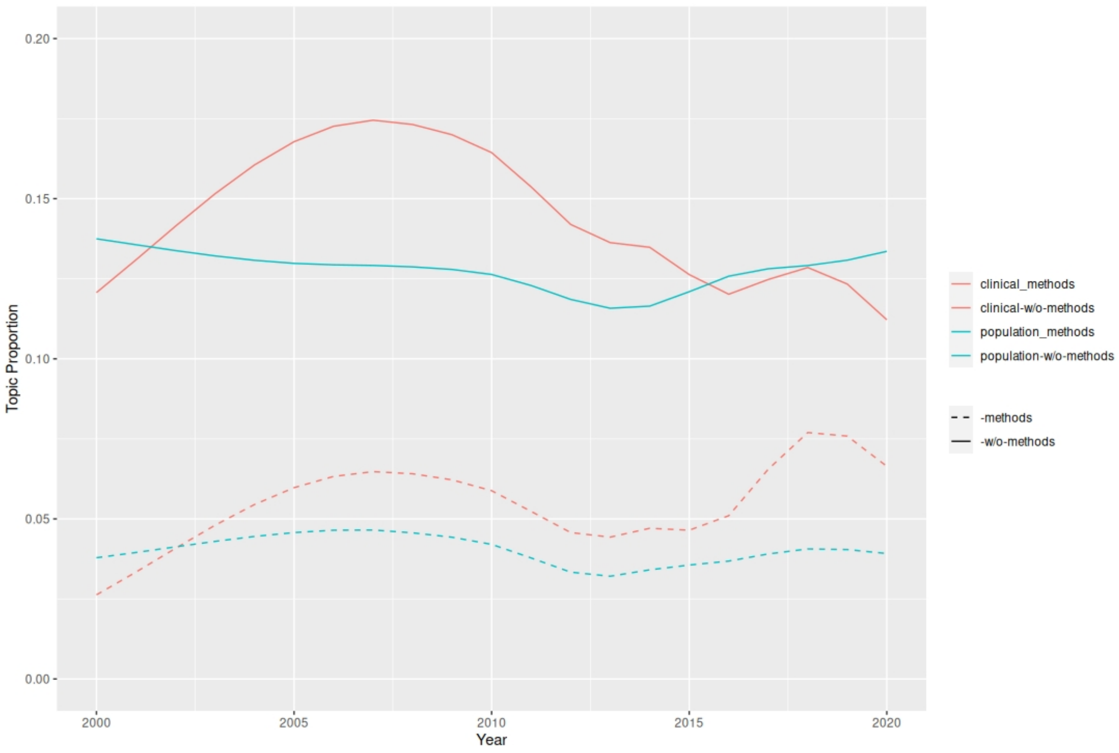

Figure B.6 Trend of the topics grouped as closer to the clinical side of epidemiology or to the population side of epidemiology isolating in both groups the topics related to methods (Bielefeld, Germany, 2024).
